# New Insights on Leucine-Rich Repeats Receptor-Like Kinase Orthologous Relationships in Angiosperms

**DOI:** 10.3389/fpls.2017.00381

**Published:** 2017-04-05

**Authors:** Jean-François Dufayard, Mathilde Bettembourg, Iris Fischer, Gaetan Droc, Emmanuel Guiderdoni, Christophe Périn, Nathalie Chantret, Anne Diévart

**Affiliations:** ^1^CIRAD, UMR AGAPMontpellier, France; ^2^INRA, UMR AGAPMontpellier, France

**Keywords:** LRR, receptor, kinase, angiosperms, phylogeny, orthologs

## Abstract

*Leucine-Rich Repeats Receptor-Like Kinase (LRR-RLK)* genes represent a large and complex gene family in plants, mainly involved in development and stress responses. These receptors are composed of an LRR-containing extracellular domain (ECD), a transmembrane domain (TM) and an intracellular kinase domain (KD). To provide new perspectives on functional analyses of these genes in model and non-model plant species, we performed a phylogenetic analysis on 8,360 LRR-RLK receptors in 31 angiosperm genomes (8 monocots and 23 dicots). We identified 101 orthologous groups (OGs) of genes being conserved among almost all monocot and dicot species analyzed. We observed that more than 10% of these OGs are absent in the Brassicaceae species studied. We show that the ECD structural features are not always conserved among orthologs, suggesting that functions may have diverged in some OG sets. Moreover, we looked at targets of positive selection footprints in 12 pairs of OGs and noticed that depending on the subgroups, positive selection occurred more frequently either in the ECDs or in the KDs.

## Introduction

Receptor-like kinases constitute one of the largest gene families in the plant kingdom. They are typically composed of an amino-terminal ECD, a TM, and an intracellular domain (ICD) containing the KD. Several phylogenetic studies of the RLK family were conducted, initially focusing on *Arabidopsis* and later including other plant species (Shiu and Bleecker, 2001b, 2003; Shiu et al., 2004; Lehti-Shiu et al., 2009; Liu et al., 2009; Sakamoto et al., 2012; Zan et al., 2013). Using a phylogeny inferred from their KD alignment the *Arabidopsis RLK* genes were classified into 44 SGs or subfamilies (Shiu and Bleecker, 2001a). Fifteen SGs have been described containing common motifs in their ECD. The ECD of the largest SG possesses LRR and this SG has therefore been named LRR-RLK (Kobe and Deisenhofer, 1994; Kajava, 1998; Shiu and Bleecker, 2001a; Shiu et al., 2004; Lehti-Shiu et al., 2009). The first members of this large family were cloned in the 90s and their signaling pathways were extensively studied. Those members are ERECTA (ER), CLAVATA1 (CLV1), BRASSINOSTEROID INSENSITIVE 1 (BRI1), SOMATIC EMBRYOGENESIS RECEPTOR-LIKE KINASE (SERK), HAESA-RLK5, and Xa21 (Horn and Walker, 1994; Song et al., 1995; Torii et al., 1996; Clark et al., 1997; Li and Chory, 1997; Schmidt et al., 1997). To date, functions have been assigned to ∼35% of the ∼230 LRR-RLK members in *A. thaliana* and – to a lesser extent – other species (Wu et al., 2016). They are important mediators of cell-cell communication to relay developmental cues and environmental stimuli or to activate defense/resistance against pathogens (Mu et al., 1994; Muschietti et al., 1998; Antolin-Llovera et al., 2014a; Belkhadir et al., 2014; Jaouannet et al., 2014).

Functional analyses conducted on *LRR-RLK* genes over the last twenty years raveled the role of the domains located in the ECD of these receptors. The LRR domains are highly versatile in number allowing a whole range of protein-protein interactions. These include homo- or hetero-dimerization of receptors, in addition to ligand binding. Furthermore, some LRR-RLK receptors possess island domains – devoid of LRRs – located between LRR motifs (Li and Chory, 1997). They were identified in the BRI1 receptor as the binding site for the brassinosteroid (BR) hormone (Kinoshita et al., 2005; Hothorn et al., 2011; She et al., 2011). Few studies have also described the functions of other ECD domains. For example, two Cys-pair have been reported. The first one is located in the N-terminal part of the LRR-RLKs, approximately 60 AA from the start codon between the SP and the first LRRs. The second one – if present – can be found between the last LRR and the TM domain (Dievart and Clark, 2003). Mutations in the Cys-pairs have been shown to affect the function of some LRR-RLKs, e.g., FLAGELLIN SENSING 2 (FLS2), a gene participating in the perception of the bacterial elicitor flagellin. However, there is also an example of a LRR receptor like protein (CLAVATA 2) for which mutations in Cys-pairs had no effect on the function of protein - at least in the meristem and roots (Noguchi et al., 1999; Song et al., 2010; Sun et al., 2012). In BRI1, a mutant harboring a mutation in Cys-pairs appears to be functional but seems to be retained in the endoplasmic reticulum and degraded. This suggests that this mutant protein does not pass the endoplasmic reticulum quality control (Hong et al., 2008). Although no general conclusions can be drawn so far on the importance of this motif, all the variations observed in Cys-pairs likely play a role in the folding, trafficking and/or the binding to other proteins. It was therefore suggested that this motif influences the signaling pathways activated downstream of the LRR-RLKs (Su et al., 2012). Another ECD, the MLD lying in between the SP and the LRRs, has also been described in one LRR-RLK SG (Hok et al., 2011). In legumes and actinorhizal plants, the SYMBIOSIS RECEPTOR LIKE KINASE (SYMRK, also known as NORK or DMI2) receptor, involved in phosphate-acquiring arbuscular mycorrhiza and in nitrogen-fixing root nodule symbiosis, possesses a malectin domain but the exact function of this receptor is still unclear (Antolin-Llovera et al., 2014a). It has been recently demonstrated that the SYMRK receptor is likely cleaved at the plasma membrane to release the N-glycosylated MLD (Antolin-Llovera et al., 2014b). Moreover, this cleavage would permit a physical interaction between the SYMRK and the LysM-type RLK NOD FACTOR RECEPTOR 5 and induces a rapid degradation of the SYMRK protein lacking its MLD. Thus, all the domains lying in the ECD with the LRRs play essential and complementary roles for specific LRR-RLK receptor functions.

Their central role in plant development and perception of environmental condition or stresses, their ubiquity in all angiosperms, and the complexity of their relationships make *LRR-RLK* genes an interesting candidate family to be studied in a phylogenetic framework (Shi et al., 2014). Such an analysis will be helpful to identify groups of orthologous genes and to compare functions between orthologs. However, inferring the phylogeny of such a large family raises several challenges. First, the vast number of sequences to be analyzed poses a problem of computational time and space. Second, the high rate of gene gains and losses during the evolution of the family, species-specific characteristics, and annotation errors result in complex orthologous relationships that are not always identified correctly by automatic gene annotation. For these reasons, large gene families – such as LRR-RLKs – are not well characterized on platforms like GreenphylDB or Phytozome dedicated to automatic clustering (Conte et al., 2008; Rouard et al., 2011; Goodstein et al., 2012) and significant manual expertise is required to produce reliable results.

In the present article, we conducted a phylogenetic analysis of the *LRR-RLK* genes from 33 plant genomes with the objective to investigate the characteristics of genes belonging to the same OGs, expected to be conserved among most monocot and dicot species analyzed. To do so, we first looked for and identified 101 OGs of genes present in most genomes analyzed defined them as the LRR-RLK angiosperm “core” sets. We observed that ECD structural features were not always conserved in some OGs, suggesting that functions may have diverged among these orthologs. We also looked at selection footprints that led to the differentiations of pairs of OGs. This allowed us to investigate the putative role and function of uncharacterized genes in recently sequenced genomes from experimentally characterized *LRR-RLK* genes in model organisms.

## Materials and Methods

### Plant Genomes Analyzed

The 33 species analyzed represent a broad spectrum of land plants (embryophyta), with one bryophyta genome, *Physcomitrella patens* (PHYPA, moss) (Rensing et al., 2008), the *Selaginella moellendorffii* genome (SELML, spikemoss) (Banks et al., 2011), representative of the lycopodiopsida, and 31 species of magnoliophyta (angiosperms), divided into eight monocot species (*Phoenix dactylifera* (PHODC, date palm) (Al-Dous et al., 2011), *Musa acuminata* (MUSAC, banana) (D’Hont et al., 2012), two subspecies of *Oryza sativa* (rice), *Oryza sativa* ssp. *japonica* (ORYSJ) (Goff et al., 2002) and *Oryza sativa* ssp. *indica* (ORYSI) (Yu et al., 2002; International Rice Genome Sequencing Project, 2005), *Brachypodium distachyon* (BRADI, purple false brome) (The International Brachypodium Initiative, 2010), *Zea mays* (MAIZE, corn) (Schnable et al., 2009), *Sorghum bicolor* (SORBI) (Paterson et al., 2009) and *Setaria italica* (SETIT, Foxtail millet)) (Bennetzen et al., 2012; Zhang et al., 2012), and 23 dicot species (two *Solanum* species, *Solanum tuberosum* (SOLTU, potato) (The Potato Genome Sequencing Consortium, 2011) and *Solanum lycopersicum* (SOLLC, tomato) (Tomato Genome Consortium, 2012), *Vitis vinifera* (VITVI, Grape Vine) (Jaillon et al., 2007), *Lotus japonicus* (LOTJA) (Sato et al., 2008), *Medicago truncatula* (MEDTR, Barrel Medic) (Young et al., 2011), *Glycine max* (GLYMA, soybean) (Schmutz et al., 2010), *Cajanus cajan* (CAJCA, pigeon pea) (Varshney et al., 2012), *Prunus persica* (PRUPE, peach) (Ahmad et al., 2011), *Malus x domestica* (MALDO, apple) (Velasco et al., 2010; Jung et al., 2012), *Ricinus communis* (RICCO, castor oil plant) (Chan et al., 2010), *Jatropha curcas* (JATCU) (Sato et al., 2011), *Manihot esculenta* (MANES, Cassava) (Prochnik et al., 2012), *Populus trichocarpa* (POPTR, black cottonwood) (Tuskan et al., 2006), two *Cucumis* species, *Cucumis sativus* (CUCSA, cucumber) (Huang et al., 2009) and *Cucumis melo* (CUCME, Muskmelon) (Sebastian et al., 2010; Garcia-Mas et al., 2012), *Schrenkiella parvula* (SCHPA (formerly EUTPR)) (Dassanayake et al., 2011), *Eutrema salsugineum* (EUTSA (formerly THEHA)) (Oh et al., 2010), *Brassica rapa* (BRARA) (Wang et al., 2011), two *Arabidopsis* species, *Arabidopsis lyrata* (ARALY) and *Arabidopsis thaliana* (ARATH, thale cress) (The Arabidopsis Genome Initiative, 2000; Hu et al., 2011), *Carica papaya* (CARPA, papaya) (Ming et al., 2008), *Gossypium raimondii* (GOSRA, cotton) (Wang et al., 2012) and *Theobroma cacao* (THECC, cacao tree) (Argout et al., 2011). Note that the genus *Thellungiella* is now known as *Eutrema*, so the species formerly known as *Thellungiella halophila* (THEHA) is now known as *Eutrema salsugineum* (EUTSA). In addition, the species of *Eutrema* sequenced at the JGI has been determined to be *salsugineum* (a close relative of *halophila*). Therefore, this genome is actually classified as *Eutrema salsugineum*. Since we started our analysis before this change, all sequences related to the *Eutrema salsugineum* genome are annotated “THEHA” or “EUTSA” in our paper. Also, in the course of our study, the name *Eutrema parvula* (EUTPR) has been changed for *Schrenkiella parvula* (SCHPA). So, all *Schrenkiella parvula* (SCHPA) sequences are annotated “EUTPR” or “SCHPA”. See Supplementary Table S1 for bibliography and web links for download. Species tree representation (Supplementary Material) is based on several studies (Koch et al., 2000; Oh and Potter, 2005; Rensing et al., 2008; Forest and Chase, 2009; Magallcdn and Castillo, 2009; Wang et al., 2009; Oh et al., 2010; Sebastian et al., 2010; Arakaki et al., 2011; Reineke et al., 2011; Wang et al., 2011, 2012; Young et al., 2011; Tomato Genome Consortium, 2012; Varshney et al., 2012; Zhang et al., 2012).

### LRR-RLKs Extraction, Phylogeny, and OGs

On each of the 33 plant proteomes, the hmmsearch program was run to extract peptide sequences containing both LRR(s) and a KD (Eddy, 2009). Sequences containing both LRRs and KD were classified into SGs using a global phylogenetic analysis (Fischer et al., 2016). First, the KD of all these sequences was aligned using MAFFT with a progressive strategy (Katoh et al., 2002). Then the alignment was cleaned with TrimAl configured to remove every sites with more than 20% of gaps or with a similarity score lower than 0.001 (Capella-Gutierrez et al., 2009). A similarity matrix was computed using ProtDist with a JTT model, and then a global distance phylogeny was inferred using FastME configured with default settings and SPR movements to optimize the tree topology (Felsenstein, 1989; Desper and Gascuel, 2002). SGs were defined manually in the global phylogeny using the *Arabidopsis* genes as reference (Shiu et al., 2004; Lehti-Shiu et al., 2009; Fischer et al., 2016). To extend this dataset to receptor kinases devoid of LRRs in their ECD (sequences annotated “No_LRR”), the BLASTP algorithm (default parameters) has been run SG per SG, using each of the 7,767 KD sequences to search a database composed of the 33 proteomes as query (Altschul et al., 1997). Blast outputs were parsed to keep only homolog sequences sharing more than 90% identity with the query sequence. The new “No_LRR” sequences retrieved by blast were assigned to the same SG as the query sequence. Then, phylogenies were inferred for each of the 20 SGs. Each group of sequences was aligned using MAFFT with an iterative strategy (maximum of 100 iterations) (Katoh et al., 2002). Alignments were cleaned using TrimAl configured this time to remove sites with more than 80% of gaps (Capella-Gutierrez et al., 2009). Then maximum likelihood phylogenies were inferred using PhyML 3.0, configured with LG+gamma model, and the best of NNI and SPR topology optimization (Guindon and Gascuel, 2003). Statistical branch supports were computed using the aLRT/SH-like strategy (Guindon et al., 2010; Anisimova et al., 2011). Each of the 20 phylogenetic trees has been reconciled with the species tree using RAP-Green (Dufayard et al., 2005)^1^. By comparing the gene tree with the species tree, this analysis allows us to root phylogenetic trees (Dufayard et al., 2005). We tested this approach of rooting (by minimizing the number of inferred duplications and losses) and compared it with rooting with outgroups (data not shown). The two methods provided very close root locations that did not change the overall conclusions.

To define the monocots dicots (MD) OGs, monocots/dicots bifurcations (branch support threshold >0.85) have been manually located in each of the 20 SG-specific trees. To be considered as MD OGs, the minimum number of monocots and dicots species represented was 3 and 4, respectively, to avoid keeping groups of misannotated sequences as MD OGs. Thus, 101 MD OGs have been defined. For each of them, the number of sequences in each of the 31 studied angiosperm species was recorded. If no sequence was discovered in one species, it was considered lost in this species.

### Structural Features

Number of motifs and positions (LRRs (PF00560.24) and KDs (PF00069.16)) are outputs of the hmmsearch program (default parameters) (Eddy, 2009). Island domains were determined based on predicted LRR positions in sequences. For SP and TM domains, the TMHMM and TOPPRED softwares have been used (Claros and von Heijne, 1994; Krogh et al., 2001). For the malectin domains in SG_I and SG_VIII-2, sequences of the domains were extracted in SMART and aligned to build hmm motifs with the hmmbuild program (Eddy, 2009; Letunic et al., 2009). For Cys-pairs, hmm motifs were built based on subsets of sequences known to possess these motifs (Eddy, 2009).

### Test of Positive Selection on Ancestral Branches

Twelve sub-trees were considered: the OGs selected were those with a ‘simple’ organization, *i.e.* with a gene topology fitting approximately with the species tree. Three to four sequences among those that are the most closely related to the OG were selected from the whole SG tree as outgroup (Supplementary Figure S4). The sequences were re-aligned and the alignment was cleaned as described previously (Fischer et al., 2014, 2016). We ran codeml branch/site models implemented in the PAML4 software (Yang, 2007). For each OG, the following branch partition was defined: all branches but one were tagged as ‘background’ branches and the branch between the duplication node and the node corresponding to the split between monocot and dicot tagged as ‘foreground’ branch. Then two models were compared: the null model (A_0_), in which sites on the fore- and background branches evolved under the same selective pressure (purifying or neutral), and a model including positive selection (model A) in which some sites on the foreground branch evolved under positive selection whereas sites on the background branches still evolved under purifying selection or neutrality. The most likely model was inferred by a likelihood ratio test (LRT). To take into account multiple testing, a Bonferroni correction was applied: the significance threshold of 0.05 was divided by the number of tested branches (24). Sites detected to be under positive selection at the codon level were manually curated for alignment quality and reliability. In branches identified to have evolved under positive selection, Bayes empirical Bayes was used to calculate the posterior probabilities at each codon and detect those under positive selection (i.e., those with a posterior probability of ω > 1 strictly above 95%).

## Results and Discussion

### More than 200 *LRR-RLK* Genes on Average Per Angiosperm Species

**FIGURE 1:**
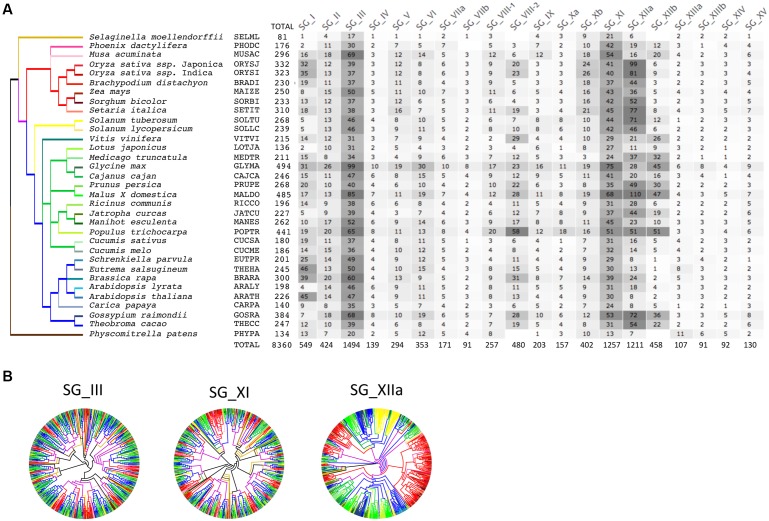
**Number and expansion of *LRR-RLK* genes in 33 plant genomes**. The species tree is based on several studies (see Material and Methods for details). Branches have been color coded to highlight monocots [red (Poaceae) and pink (others)] versus dicots [yellow (Solanaceae), green (Eurosids I) and blue (Eurosids II)] species. *Selaginella* and moss species, are respectively, in light and dark brown. **(A)** Numbers of *LRR-RLK* genes found in each proteomes and each SG are indicated. The gray coloring, from light to dark, is proportional to the number of genes found. **(B)** Unrooted phylogenetic trees of the three biggest SGs. Note the different expansion patterns in SG_III and SG_XI vs. SG_XIIa.

We conducted a phylogenetic analysis of the *LRR-RLK* gene family in 33 fully sequenced plant genomes to classify them into SGs and to highlight and describe general characteristics of these LRR-RLK gene sets (Fischer et al., 2016). Briefly, besides 31 angiosperms genomes – represented by eight monocots (including six poaceae) and 23 dicots – one bryophyte genome of *Physcomitrella patens* (PHYPA, moss) and one lycopodiopsida genome of *Selaginella moellendorffii* (SELML, spikemoss) were included (Supplementary Table S1, see Section “Materials and Methods” for details and five-digit species code). As it has been done previously, we based our classification of *LRR-RLK* genes into SGs on the KD phylogeny (Shiu et al., 2004; Lehti-Shiu et al., 2009; Fischer et al., 2016). In our previous study (Fischer et al., 2016), the LRR-RLK dataset contained 7,767 sequences possessing at least one LRR in their ECD. Since we focus on structural features of ECDs and presence/absence of the genes in LRR-RLK OGs in this present study, we included LRR-RLK homologues for which LRRs were completely lost or were degenerated in this new dataset. Thus, 593 sequences (prefixed “No_LRR”) were added to the original set of 7,767 sequences which lead to a total of 8,360 sequences (Supplementary Table S2). Within each of the 20 SGs, KDs were aligned and SG-specific trees were obtained using a likelihood-based method (PHYML) (**Figure 1A** and Supplementary Material). Altogether, these *LRR-RLK* genes represent on average 0.71 and 0.66% of the monocot and dicot proteomes, respectively. Interestingly, in moss (PHYPA) and spikemoss (SELML), the proportions of LRR-RLKs per genome (0.41 and 0.36%, respectively) are approximately half the ratio observed in angiosperms. Likewise, the average number of LRR-RLK genes in angiosperms is 263.6, with 260.7 LRR-RLK proteins [±20.2 (SE)] in dicots and 268.8 [±18.2 (SE)] in monocots. In PHYPA and SELML, 134 and 81 *LRR-RLK* genes have been retrieved, respectively. There is no significant difference in the average number of *LRR-RLK* genes between monocots and dicots but the number almost doubled in most angiosperms compared to PHYPA and SELML. It has to be noted that in some genomes (e.g., CARPA and LOTJA), the number of *LRR-RLK* genes is particularly low compared to other angiosperm genomes, suggesting that retention rates vary among genomes, and that many losses may have occured in some genomes (Fischer et al., 2016). Nevertheless, our results highlight the fact that, after the first wave of expansion in early land plants (Embryophyta), a second large amplification occured in angiosperm genomes which shaped the current LRR-RLK family size of more than 200 gene copies on average per genome.

Among the 20 SGs, SG_III, SG_XI, and SG_XIIa are the largest as they contain ∼50% of the total number of *LRR-RLK* genes in the analyzed plant genomes (**Figure 1A**). The extensive expansions leading to their size do not follow the same amplification pattern (**Figure 1B**). This observation is highlighted in **Figure 1B** by the color code used for each species in the 20 SG-specific trees (with branches of monocots species in pink and red, and branches of dicots species in yellow, blue and green). Our results reveal that the high numbers of SG_XIIa genes is the consequence of many lineage-specific expansions (LSE) (See Fischer et al., 2016 for details). These LSEs are relatively recent as they can be observed in phyla as well as species-specific lineages. On the contrary, in SG_III and SG_XI, expansions occured mainly before the early divergence of angiosperm lineages, even though LSEs can also be observed at different levels of resolution in the trees. Therefore, these numerous and diverse modes of expansions lead to complex paralogous and orthologous relationships.

### 101 OGs of Monocot and Dicot Genes Retained Along Angiosperm Evolution

**FIGURE 2:**
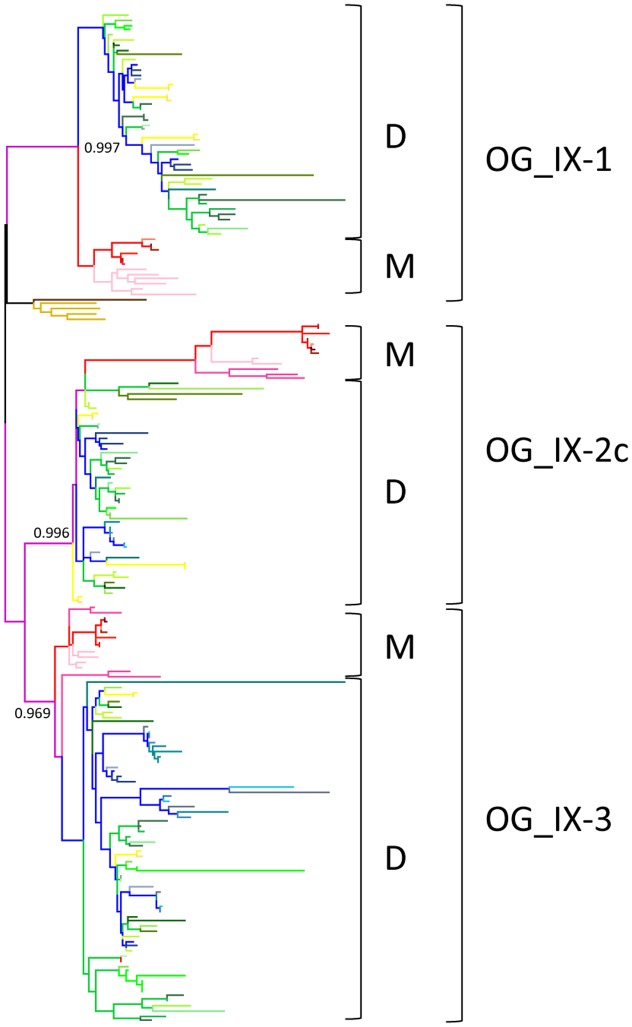
**Example of monocots/dicots OG characterization in SG_IX**. Branches are color coded according to **Figure 1** with branches of monocots (M) species in pink and red, branches of dicots (D) species in yellow, blue and green, and branches of moss and spikemoss in light and dark brown, respectively. OGs containing M and D genes are represented as MD OGs and numbered by SG. Within these OGs, the orthologous relationships can be “simple” or “complex” (OG_c). Note that the presence and number of paralogs after monocots and dicots divergence is not taken into account. Numbers at monocots/dicots bifurcations represent nodes with statistical branch supports aLRT/SH-like >0.85.

With the aim of transfering functional annotation from well studied genes from model species to orthologous genes in other genomes, we first analyzed in depth orthologous relationships between monocots and dicots *LRR-RLK* genes in each SG. This analysis led us to define what we named the “core set” of *LRR-RLK* genes in angiosperms: i.e., orthologous genes which have not been completely lost in either monocots or dicots throughout the angiosperms evolutionary history. To do so, the 20 SG-specific trees were scaned to locate monocots/dicots bifurcations (**Figure 2**). Based on this analysis, 101 OGs containing monocots and dicots sequences (named MD OGs) were characterized and defined as the “core” set of *LRR-RLK* genes in angiosperms.

**FIGURE 3:**
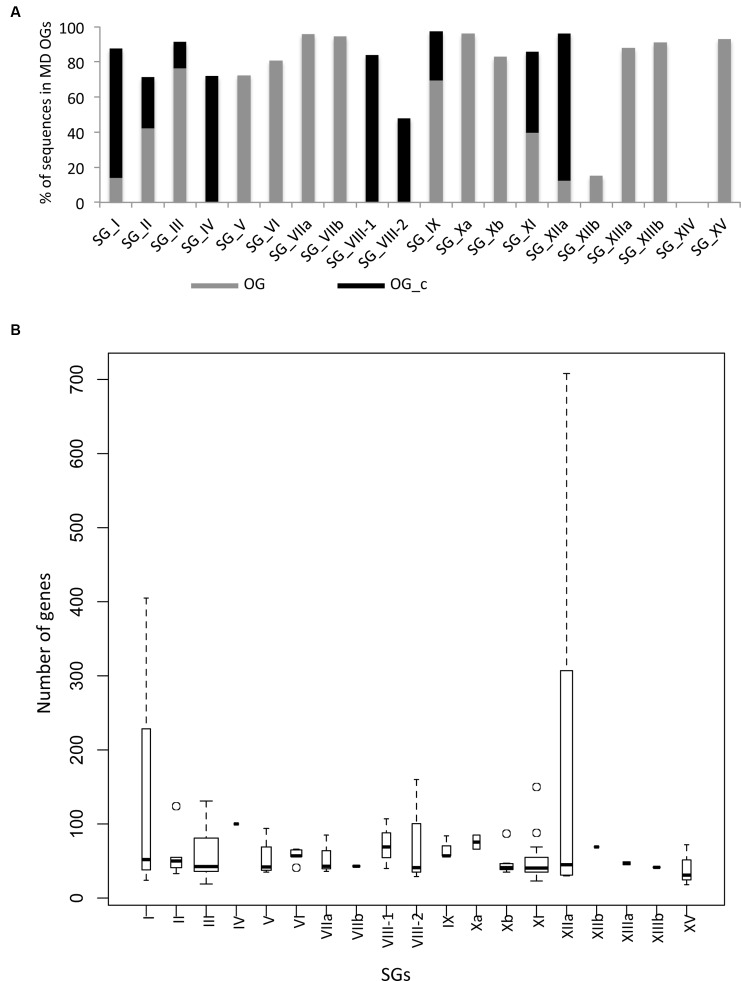
**Distribution of genes per OGs and per SGs. (A)** Percentage of genes contained in non complex (OG) and complex OGs (OG_c). **(B)** Number of genes per OGs. The width of boxes are proportional to the number of OGs. Note that SG_III and SG_XI are the largest.

The SG analysis revealed that these 101 MD OGs are present in 19 of the 20 SGs, with the majority of them in SG_III and SG_XI (**Table 1**). This highlights again the fact that most of the *LRR-RLK* genes which underwent expansions before the monocots/dicots split have been retained in these SGs. In order to go further in the description of orthologous relationships, we qualified OGs as either “simple” or “complex”. In “simple” OGs, the presence or absence of duplications within the monocot or dicot clades can clearly be inferred from the phylogenetic tree. On the other hand, if several duplications occurred disorderly with no obvious connection to the species tree we described these OGs as “complex” (OG_c). Interestingly, these OG_c are over-represented in SG_IV, VIII-1, VIII-2, and XI. A total of 6739 genes are contained in the 101 OGs, representing 82.7% of the entire *LRR-RLK* gene family. However, while 2956 genes are included in the 24 OG_c (average of 123.2 genes per OG), 3783 genes belong to the 77 non-complex OGs (average of 49.2 genes per OG), highlighting differences in expansion/retention rates between these OGs. Moreover, looking at the percentage of genes contained in OGs per SG reveals that, except for SG_VIII-2 and XIIb, more than 70% of the *LRR-RLK* genes belong to OGs, with genes mainly in complex OGs in SG_I, IV, VIII-1, VIII-2, XI, and XIIa (**Figure 3A**). In some SGs, some OGs contain a very large number of genes, such as 405 genes in one of SG_I OG (SG_I-3c), or 307 and 708 genes in two SG_XIIa OGs (**Figure 3B** and Supplementary Table S2 for details). In these large OGs, many species-specific duplications occured, and among the 10 OGs containing more than 100 genes, 8 are complex. In SG_I-3c for example, several genes have been studied in *Arabidopsis*, such as IMPAIRED OOMYCETE SUSCEPTIBILITY 1 (IOS1), FLG22-INDUCED RECEPTOR-LIKE KINASE 1 (FRK1), and light-repressible receptor protein kinase (LRRPK) (Deeken and Kaldenhoff, 1997; Asai et al., 2002). All have been reported to be involved in abiotic and biotic responses in dicots but no gene from the same OG have been described so far in monocots. However, the fact that these genes are classified into the complex mode of expansion suggests that in monocots too, these genes could be involved in stress response.

**Table 1:** Number of MD OGs per SG.

SG	Total number of MD OG	Number of OG_c
SG_I	3	1
SG_II	5	1
SG_III	24	3
SG_IV	1	1
SG_V	4	
SG_VI	5	
SG_VIIa	3	
SG_VIIb	2	
SG_VIII-1	3	3
SG_VIII-2	3	3
SG_IX	3	1
SG_Xa	2	
SG_Xb	7	
SG_XI	22	9
SG_XIIa	6	2
SG_XIIb	1	
SG_XIIIa	2	
SG_XIIIb	2	
SG_XIV	0	
SG_XV	3	
**Total**	101	24

### More than 10% of the LRR-RLK Core Sets Are Absent in Brassicaceae

**FIGURE 4:**
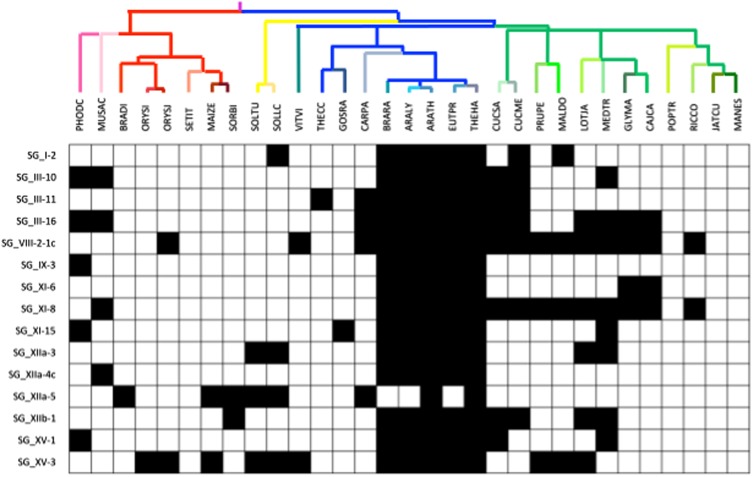
**Presence/absence of OGs in the *Arabidopsis* clade**. Presence/absence is represented by white/black boxes, respectively.

For each OG, we investigated whether some species were lacking members, focusing particularly on the Brassicales, for which six species are included in our analysis (**Figure 4**). Moreover, this clade contains the model plant *Arabidopsis* which is the reference for many studies on LRR-RLK functions (Wu et al., 2016). Interestingly, among the 101 OGs, 14 (13.8%) have been completely lost in the Brassicaceae, and 3 of them are even absent in all the Brassicales. This observation is an incentive to the extensive study of these receptors in other plants than *Arabidopsis*, adding an argument to the fact that functions or interactions are sometimes phylum-specific. For example, the SG_I-2 OG contains the SYMBIOSIS RECEPTOR LIKE KINASE (SYMRK, also known as NORK or DMI2) receptor which is involved in actinorhizal plants and legumes, respectively, in phosphate-acquiring arbuscular mycorrhiza and nitrogen-fixing root nodule symbiosis (Antolin-Llovera et al., 2014a). For this gene, we noticed that besides being absent in the Brassicaceae, which do not form mycorrhizal associations and root nodule symbiosis with rhizobia, some other characteristics of these receptors have been observed in monocots (see below).

### Inference of Functional Information from Experimentally Characterized *LRR-RLK* Genes to Uncharacterized Genes

**FIGURE 5:**
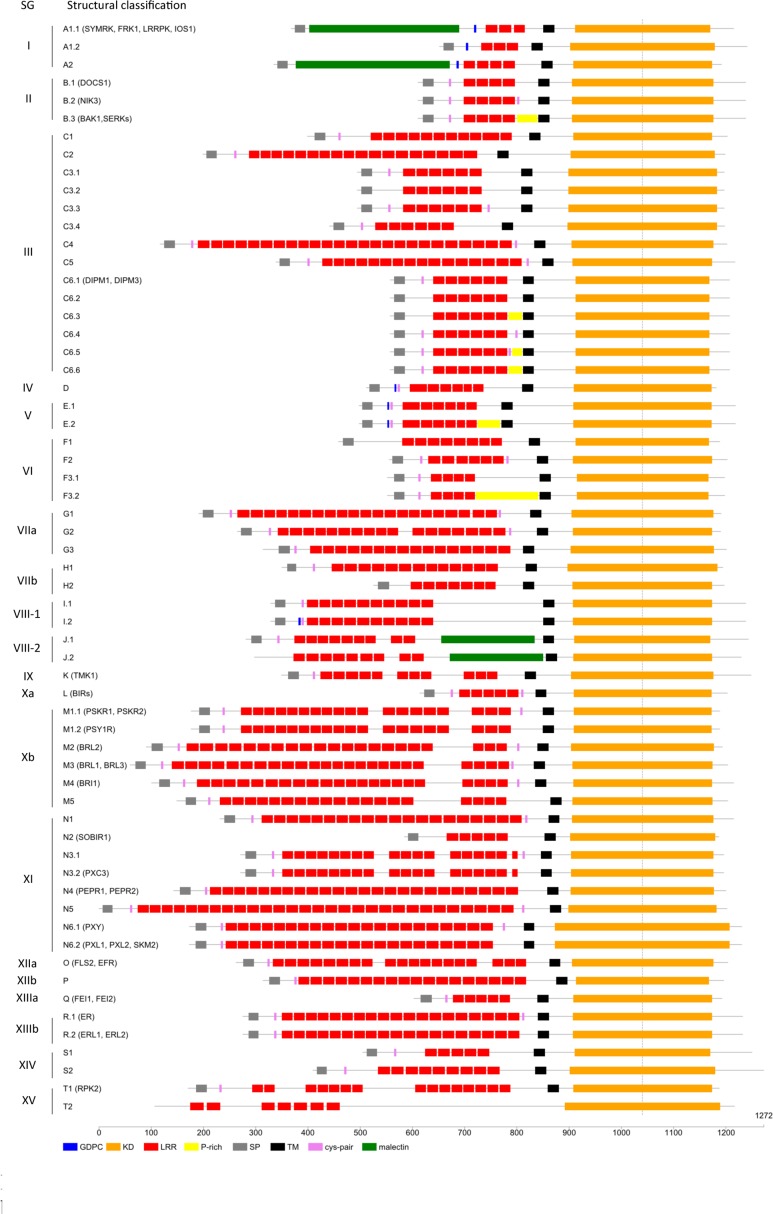
**Leucine-Rich Repeats Receptor-Like Kinase structural features**. Schematic representation of the subdivided SGs based on ECD characteristics with names of *LRR-RLK* genes discussed in the article.

The use of orthologous relationships to infer functional annotations relies on the fact that orthologs are expected to carry equivalent functions in different organisms. However, this can only be reliably inferred if, at least, structural characteristics and domain architecture are conserved between othologs. In our analysis, the phylogeny of the LRR-RLK proteins was computed on the well conserved KDs. However, LRR-RLK sequences are composed of several domains and, notably, of LRRs in their ECD. One could wonder whether the domains belonging to genes of the same OG are conserved. First, we took a detailed look at the predicted number of LRRs of all these receptors. The number of LRR motifs per protein is an important feature for homo- and hetero-complex formation between LRR-RLKs (Macho and Zipfel, 2014). Second, we investigated the presence of island domains in between LRRs. These domains have been described to be the binding site for the BR hormone in some receptors (Kinoshita et al., 2005; Hothorn et al., 2011; She et al., 2011). Third, we analyzed the presence of the MLD, a carbohydrate-binding domain, and the GDPC, a protein cleavage motif, which were shown to be located before the LRRs in some SG_I receptors. Fourth, we investigated the presence of Cys-pairs surrounding the LRRs in some SGs. The presence and organization of these domains is functionally important and has to be taken into account for transfering functional informations between orthologous genes. The description of structural features localized in the ECDs of these receptors allows a subclassification which, although reflecting the phylogeny of the KD of these receptors, also takes the structural differences of the ECD into account. Therefore, we subdivided the 20 SGs further according to these characteristics in their ECD (**Figure 5** and Supplementary Table S3 for details).

#### Number of LRR Motifs

**FIGURE 6:**
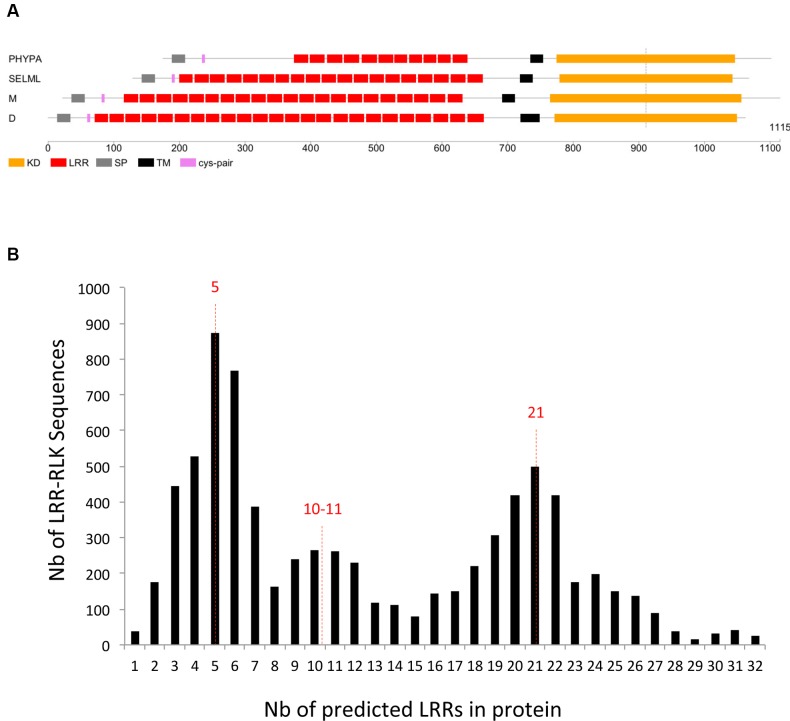
**Leucine-Rich Repeats motifs. (A)** Schematic representation of the FLS2 orthologs in moss (PHYPA), Selaginella (SELML), and monocots (M) and dicots (D). Note that the number of predicted LRR motifs in PHYPA is lower than in other phyla. **(B)** Number of predicted LRRs per protein. The majority of the sequences contain around 5, 10, or 21 LRRs.

First, for the moss and/or spikemoss genes which are orthologous to the angiosperm core sets of genes, we investigated if some sets of receptors varied in the number of LRR motifs. All those genes have a common ancestor, predating the divergence between moss and/or spikemoss and angiosperms and their KDs have all evolved in concert for ∼450 MYA. Despite speciation events, the close phylogenetic relationship of all these *LRR-RLK* genes with moss and spikemoss orthologs suggests that signaling pathways downtream of these receptors could be conserved. In the OG containing the FLS2 receptor (SG_XIIa), we noticed that the number of LRRs in the PHYPA orthologs was lower than in angiosperms (**Figure 6A**). This peculiar differences noted in the PHYPA ECDs of the FLS2 orthologs could affect ligand binding or even suggest that ligands are not conserved. This would be in agreement with publications stating that the moss *Physcomitrella patens* does not carry an FLS2 ortholog and also shows no response to flg22 (Boller and Felix, 2009; Tanigaki et al., 2014). All other core gene sets for which *Physcomitrella*/*Selaginella* ECDs are conserved compared to angiosperms, provide interesting cases for which it would be worth to verify if the functions described for monocots and/or dicots members are entirely conserved in bryophytes and lycopsids.

Second, we focused on the number of predicted LRRs in the 7,767 LRR-containing sequences. Even if the number of LRRs per sequence is very variable, the distribution of the number of LRR per sequences shows three peaks at 5, 20, or 21 (**Figure 6B**). This observation suggests that these numbers of LRRs per sequences may be optimal for the 3D conformation of these receptors and their interactions in homo- or heterocomplexes. To our knowledge, this observation has not been explicitly made in animal LRR-containing proteins but could also be true (Ng et al., 2011). In plants, one hetero-oligomeric protein complex has been described between the BR receptor BRI1 (SG_Xb M4 in **Figure 5**) and BAK1/SERK3 or SERK1 receptors (SG_II B.3 in **Figure 5**) (Aker and de Vries, 2008; Chinchilla et al., 2009; Santiago et al., 2013; Sun et al., 2013). The complex crystal structure of SERK1 and BRI1 has revealed that the BRI1 C-terminal LRRs form a docking platform for the LRRs of the SERK1 co-receptor (Hothorn et al., 2011; Santiago et al., 2013). The SERK proteins have also been shown to serve various other BR-independent functions by forming heterocomplexes with SG_XIIa receptors (FLS2 and EFR, structure O in **Figure 5**) and the PEP1 RECEPTOR proteins (PEPR1, SG_XI N4 in **Figure 5**) (Chinchilla et al., 2007; Heese et al., 2007; Albrecht et al., 2008; Schulze et al., 2010; Roux et al., 2011; Koller and Bent, 2014). In rice, OsSERK2 (SG_II B.3 in **Figure 5**) forms a constitutive complex with the LRR-RLK Xa21 (SG_XIIa O in **Figure 5**) (Chen et al., 2014). Thus, these SERK co-receptors (4-5 LRRs) seem to play a central role in the regulation of multiple LRR-RLKs (>20 LRRs) by interacting directly with them (Aker and de Vries, 2008; Chinchilla et al., 2009; Kim et al., 2013; Santiago et al., 2013; Sun et al., 2013). Interestingly, SERK3/BAK1 has also been found in complex with the BAK1-INTERACTING RECEPTOR KINASE1 and 2 (BIR1 and BIR2) proteins, two receptors belonging to SG_Xa, another SG possessing five LRRs in its ECD (structure L in **Figure 5**) (Gao et al., 2009; Halter et al., 2014). The association between SERK3/BAK1 and BIR1/2 prevents the FLS2-BAK1 interaction before elicitation of the immune response. It is still unknown if the co-receptor status is restricted to the SERK genes subfamily of SG_II B.3, or if other LRR-RLK SGs could also be part of various signaling pathways in interaction with receptors possessing 20-25 LRRs. Indeed, other SGs such as SG_XI, SG_XIIIa, and SG_XIV corresponding to structures N2, Q and S1, respectively (**Figure 5**), also possess five LRRs in their ECD. In SG_XI-22 (structure N2 in **Figure 5**), the receptor SUPPRESSOR OF BIR1 1 (SOBIR1) also named EVERSHED (EVR) has been shown to be involved in floral organ shedding and in the regulation of several resistance signaling pathways with the BIR1, BAK1, and FLS2 receptors (Gao et al., 2009; Leslie et al., 2010). Moreover, the SOBIR1 receptor is also described as a coreceptor/adaptor forming complexes with many LRR-Receptor like proteins (LRR receptors devoid of a KD), suggesting that the SERK-type receptors (5-LRRs) could be considered as general adaptors important for functionality in complex with their receptor partners (Liebrand et al., 2013; Gust and Felix, 2014). In SG_XIIIa (structure Q), the FEIs receptors (FEI1 and FEI2, named after the Chinese word for fat), whose single mutants were indistinguishable from the wild type in development, work against a co-receptor function (Xu et al., 2008). However, for other SGs possessing five LRRs in their ECD, the question about the putative co-receptor function will remain unanswered until further molecular characterization is performed. The receptors belonging to SG_II B.3, contrary to SG_II B.1 and B.2, contain also a Pro-rich motif in their ECD. The question on the functionality of the Pro-rich domain in the ECD of the SERK proteins also remains to be answered. This domain of unknown function could provide a flexible hinge to the ECD (Schmidt et al., 1997; Kay et al., 2000; Baudino et al., 2001; Hecht et al., 2001; Chevalier et al., 2005). Interestingly, other SGs possess these kinds of motifs, e.g., SG_VI F3.2, for which no receptor has been studied yet.

#### Island Domains

We positioned all the predicted LRRs on the 7,767 proteins and searched for islands between them (Supplementary Table S4). These islands are of particular importance as they are the brassinolide hormone binding sites for the BRI1 and BRI1-like (BRL) receptors (structures M2–M4 in **Figure 5**) belonging to SG_Xb (Hothorn et al., 2011; She et al., 2011). We found that in SG_Xb, all sets of orthologs possess an island encompassing two (Structures M1 in **Figure 5**) or at least three (structures M2–M5) LRRs. One could ask if the island domain in genes of structure M5, for which no function has been described up to now and very similar to the M2–M4 structures, is also a binding site for the BR hormone. The remaining OGs in SG_Xb (structure M1) contain the three *Arabidopsis* genes: *PSKR1, PSKR2*, and *PLANT PEPTIDE CONTAINING SULFATED TYROSINE 1 RECEPTOR (PSY1R)*. These receptors have overlapping functions in promoting cellular proliferation, longevity and expansion (Matsubayashi et al., 2006; Amano et al., 2007; Hartmann et al., 2013). The PSKR subfamily is also required for PSK peptide signaling in sexual reproduction in plants (Stuhrwohldt et al., 2015). Moreover, these receptors play a role in modifying responses to biotic pathogens and wounding (Loivamaki et al., 2010; Mosher et al., 2013). In the three *Arabidopsis* PSKRs, one island of ∼60 AA was detected in addition to other smaller ones specific to each receptor. These islands could be important for hormone binding like in BRI1 and BRL receptors (Hothorn et al., 2011; She et al., 2011). Indeed, it has been shown that the BR hormone could play a role in the signaling pathways activated downstream of these receptors (Hartmann et al., 2013). In SG_IX (structure K) and SG_XV (structure T1), islands encompassing the size of at least two LRRs are also present. In SG_IX, the crystal structure of the *Arabidopsis* TRANSMEMBRANE KINASE 1 [TMK1, also known as BLK1 (BARK1-like Kinase 1)] suggests that the islands could be critical for structural integrity. In SG_XV, the crystal structure of RECEPTOR-LIKE PROTEIN KINASE 2 (RPK2, also known as TOAD2) suggests that the islands could be the site for ligand binding as in BRI1 (Liu et al., 2013; Song et al., 2014).

#### Additional Domains

As mentioned previously, a MLD lying in between the SP and the LRRs has been described in some SG_I receptors (**Figure 5**) (Hok et al., 2011; Antolin-Llovera et al., 2014a). One of them is the SYMRK receptor (structure A1.1) involved in mycorrhizal associations and rhizobium-legumes symbiosis, but its exact function is still unclear (Antolin-Llovera et al., 2014a). Recently, it has been demonstrated that the SYMRK receptor is cleaved at a GDPC motif placed at the end of the MLD to release the N-glycosylated ectodomain in the absence of symbiotic stimulation (Antolin-Llovera et al., 2014b). Moreover, protein cleavage on this motif would permit a physical interaction with the LysM-type RLK NOD FACTOR RECEPTOR 5 (NFR5) and induces a rapid degradation of the SYMRK protein lacking its MLD. In this form, SYMRK could act as a co-receptor to initiate symbiotic signaling with NFR5 and would mirror the role played by the receptors of the SERK family. After MLD release, the structure of SYMRK could indeed resemble that of BAK1/SERK3. Our analysis of structural features of ECDs reveals that the monocot orthologs of the SYMRK receptor were much shorter than the dicot ones and that the monocot receptors are devoid of the MLD present in dicots (structure A1.2). Thus, the SYMRK activation mechanism will have to be further investigated in monocots to evaluate if it can fit into the dicots model, or if other receptors possessing a MLD (SG_I or others, see below) are involved in this process.

We therefore looked for malectin domains in all LRR-RLK sequences and found that SG_VIII-2 receptors also contain one. However, in SG_VIII-2, this domain is not located just after the SP but between the LRRs and the TM domain (Structure J in **Figure 5**). We also found the GDPC cleavage motif in most of the SG_IV, V and SG_VIII-1 receptors. However, contrary to SG_I, the GDPC site is located just before the first Cys-pairs in all other SGs. In these SGs, the Cys of the GDPC motif is the first site of the Cys-pair. It is still unknown if these receptors are also cleaved at this site and what the functional consequences would be. In SG_VIII-2, which contains a malectin domain C-terminal of the LRRs, no GDPC sites are present. This does not exclude the possibility that another cleavage site could be used to truncate the protein. Thus, the function of the malectin domains in SG_VIII-2 will have to be explored in the future to decipher their exact functional role and their potential involvement in protein stabilization.

### Positive Selection in the Divergence between Ancestrally Duplicated OGs

Twelve pairs of MD OGs present in almost all monocot and dicot species, and harboring a gene topology fitting approximately the species tree, appear to be issued from ancestral duplications predating the monocot/dicot divergence (**Table 2**). As these OG pairs had a similar ECD structural organization and were kept in almost all species studied here, we searched for potential positive selection footprints in the divergence leading to their differentiation. Although these genes do not all have a known function, it is expected that they underwent amino-acid changes leading to their sub- and/or neo-functionalization, and one can wonder if and how positive selection could have driven these changes.

**Table 2:** Positive selection on branches of pairs of OGs.

SG	Number of sequences	Name of branch	Structural SG	Known genes	Model	Number of parameters	lnL	*P*-value		Number of sites	ECD	ICD
SG_II	101	SG_II-3	B.1	DOCS1	A_0_	203	-47756.49038	0.2960935	ns	-		


					A	204	-47755.94453					


		SG_II-4	B.2	NIK3	A_0_	203	-47761.1425	0.0012474	^∗^	3	3	


					A	204	-47755.93314					


SG_III	92	SG_III-3	C2		A_0_	185	-87752.00789	0.0013461	^∗^	1	1	


					A	186	-87746.86878					


		SG_III-4	C2		A_0_	185	-87749.51178	0.0000735	^∗∗^	0		


					A	186	-87741.65272					


SG_III	126	SG_III-9	C6.1	DIPM1, DIPM3	A_0_	253	-58707.08864	0.000000	^∗∗^	11	4	7


					A	254	-58683.61908					


		SG_III-8	C6.2		A_0_	253	-58712.53117	0.000000	^∗∗^	5	1	4


					A	254	-58695.21242					


SG_Xa	145	SG_Xa-1	L	BIR1	A_0_	291	-80168.41936	0.013983288	ns	-		


					A	292	-80165.39924					


		SG_Xa-2	L	BIR2, BIR3, BIR4	A_0_	291	-80166.7335	0.031737545	ns	-		


					A	292	-80164.42719					


SGXb	162	SG_Xb-5	M1.1	PSKR2	A_0_	325	-148131.1793	0.000381394	^∗∗^	1	1	


					A	326	-148124.8687					


		SG_Xb-6	M1.1	PSKR1	A_0_	325	-148131.1246	0.000004	^∗∗^	1	1	


					A	326	-148120.4449					


SG_XI	99	SG_XI-20	N3.2	PXC3	A_0_	199	-40840.3843	0.00030735	^∗∗^	0		


					A	200	-40833.87176					


		SG_XI-19	N3.2		A_0_	199	-40838.95437	0.000838	^∗^	1	1	


					A	200	-40833.37719					


SG_XI	116	SG_XI-9	N4	PEPR1, PEPR2	A_0_	233	-80676.72267	9.80354E-09	^∗∗^	12	8	4


					A	234	-80660.28275					


		SG_XI-8	N4		A_0_	233	-80679.22227	0.000000	^∗∗^	9	3	6


					A	234	-80660.42399					


SG_XI	115	SG_XI-14	N6.2	SKM2	A_0_	231	-126354.8399	3.48134E-19	^∗∗^	20	12	6


					A	232	-126314.768					


		SG_XI-15	N6.1		A_0_	231	-126374.0677	0.000000	^∗∗^	12	8	3


					A	232	-126335.0153					


SG_XI	132	SG_XI-18	N6.1	PXY	A_0_	265	-131270.9444	1.13374E-34	^∗∗^	5	4	1


					A	266	-131195.5224					


		SG_XI-17	N6.2	PXL1, PXL2	A_0_	265	-131261.1148	2.96009E-40	^∗∗^	23	14	9


					A	266	-131172.9143					


SG_XIIa	101	SG_XIIa-2	O		A_0_	203	-69490.32865	0.020062	ns	-		


					A	204	-69487.62542					


		SG_XIIa-3	O		A_0_	203	-69490.53986	0.047369	ns	-		


					A	204	-69488.57373					


SG_XIIIa	143	SG_XIIIa-2	Q		A_0_	287	-66690.11714	0.006790	ns	-		


					A	288	-66686.45331					


		SG_XIIIa-1	Q	FEI1, FEI2	A_0_	287	-66684.64365	0.000737	^∗^	0		


					A	288	-66678.94678					


SG_XIIIb	143	SG_XIIIb-1	R.2	ERL1, ERL2	A_0_	161	-79161.61197	3.21923E-09	^∗∗^	26	14	8


					A	162	-79144.08871					


		SG_XIIIb-2	R.1	ER	A_0_	161	-79156.50926	0.000012	^∗∗^	11	8	3


					A	162	-79146.91448					

*lnL, log-likelihood; *P*-values, ns, Likelihood ratio test not significant; ^∗^, significant at 5%-level; ^∗∗^, significant at 1%-level after the Bonferroni correction (see Matreials and Methods)*.

To answer this question, we tested whether some sites underwent positive selection on the two branches starting from the ancestral duplication and ending at the monocot/dicot divergence node of each OG. The detailed results of this analysis are presented in **Table 2** and Supplementary Table S5. Two pairs showed no signal on either of the two branches (SG_Xa-1/2, SG_XIIa-2/3). For the pairs SG_II-3/4 and SG_XIIIa-1/2, a signal was detected for one branch only but the signal on SG_XIIIa-1/2 may be a false positive or the sign of a lack of power, since no sites appeared to be significant (see Materials and Methods for details). The eight other pairs showed a signal of positive selection on each branch. Although the model indicating positive selection performs significantly better than the null model, two pairs (SG_III-3/4 and SG_XI-19/20) have no significant sites for one of the two tested branches. This again indicates either a false positive or a lack of power. It is also possible that positive selection acted on a large number of sites which results in none of them exceeding the significance threshold. Finally, five pairs (SG_III-8/9, SG_XI-8/9, SG_XI-14/15, SG_XI-17/18, and SG_XIIIb-1/2) have a strong signal with up to 26 sites validated after manual curation. This result shows that in about half of the tested cases, several amino acid changes fixed in the divergence between these genes are compatible with a signal of positive selection.

**FIGURE 7:**
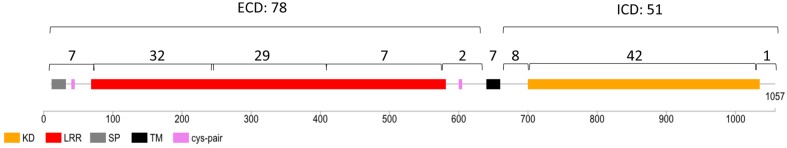
**Number of sites under positive selection in SG_III, XI, or XIIIb per domain**. Schematic representation of a LRR-RLK receptor containing around 20 LRRs.

A total of 141 sites were manually validated as having experienced an episode of positive selection during MD OG genes divergence. For the five pairs with a strong signal, the repartition of these sites across the different domains of the LRR-RLK protein showed that the LRRs and KDs are the most affected (**Figure 7**). More than half of the sites (78) fall in the ECD, among which 68 are in the LRR domain; 51 sites fall in the ICD, among which 42 are in the KD (**Table 2**). Considering that these LRR and KD are the largest domains, we normalized the number of positively selected sites by domain size. Kinase and LRR appeared then to be affected equally (Chi-square test, *p* = 0.26) by positive selection. This result is very different from positive selection signatures observed in the recent paralogs, for which LRR is the most strongly affected domain (Fischer et al., 2016). The number of sites laying in the LRR domain allowed us to look for any specific distribution across the 24 amino acids composing the motif. The repartition of the sites affected by positive selection within the LRR is not homogeneous and the majority of them (67) fall in the 13 non-canonical positions (Supplementary Table S5). However no notable pattern emerges from their distribution (data not shown). Again, this result contrasts with what is observed in lineage-specific expanded genes for which four positions are predominantly affected (Fischer et al., 2016). The remaining domains are affected by a number of sites varying from 1 to 9.

This approach revealed a prevalence of sites targeted by positive selection in the ECD for the three couples of genes belonging to SG_XI as well as SG_XIIIb. On the opposite, a tendency to target ICDs can be observed for SG_III. Two pairs of OGs for which positive selection footprints are detected in the ECD correspond to OGs whose duplication gave rise to the PXY and PXLs clades in SG_XI-17/18 (Fisher and Turner, 2007; Jung et al., 2015); and to the ERECTA (ER) and ERECTA-like (ERL) clades in SG_XIIIb-1/2 (Torii et al., 1996; Sanchez-Rodriguez et al., 2009). Other pairs of OGs concern differentiation of the PEPR1 and 2 clade (SG_XI-9), of the STERILITY-REGULATING KINASE MEMBER 2 (SKM2) gene (SG_XI-14) or of the DspA/E-interacting protein of Malus x domestica Borkh 1 and 3 (DIPM1 and 3) clade (SG_III-9), from their respective sister clades, SG_XI-8, SG_XI-15, and SG_III-8 (Meng et al., 2006; Krol et al., 2010; Kang and Hardtke, 2016). In these clades, no gene has been described yet. The strong signal of positive selection detected for these five groups of genes indicates that the divergence between ancestral copies may have procured a selective advantageous: in the domain involved in ligands or partners binding for ECDs, or in the domain affecting downstream signaling pathways for ICDs. Indeed, during the early expansion of LRR-RLK that took place before angiosperm split, some duplicated LRR-RLK differentiated by fixation of a higher number of non-synonymous than synonymous mutations at some amino acid sites, indicating the emergence of probably new advantageous functions.

## Conclusion

In this report, we provide a framework to aid in the classification and give new insights to new prospects for functional analysis of some plant LRR-RLKs. We have defined the “core set” of the large *LRR-RLK* gene family and classified these receptors based on their ECD features. These analyses reveal that even if the KDs of the LRR-RLKs are phylogenetically related, the ECDs may have been subjected to major (e.g., loss of LRRs revealed by the structural features characterization) or minor (e.g., point mutations revealed by the traces of positive selection analysis) modifications during the evolution of orthologs. These alterations could affect ligand recognition sites, dimerization with other receptors, and/or other processes involved in signal transduction. Indeed, the proper signal transduction via receptor kinases is not restricted to the binding of ligands to receptors located at the plasma membrane. Tightly regulated steps for proper folding of the proteins, trafficking from endomembranes to plasma membranes, and finally internalization and recycling of the receptors after ligand binding play essential roles in signal transduction (Shah et al., 2002; Robatzek et al., 2006; Salomon and Robatzek, 2006; Irani and Russinova, 2009; Beck et al., 2012; Di Rubbo et al., 2013; Offringa and Huang, 2013; Martins et al., 2015). Recently, an enthusiastic wave swept over the plant receptor kinases community concerning endoplasmic reticulum quality control since most of these steps take place in this cellular compartment (Saijo, 2010; Su et al., 2011; Huttner and Strasser, 2012; Tintor and Saijo, 2014). Newly synthetised membrane-resident proteins translocate first into the endoplasmic reticulum where they are subjected to folding and modifications like formation of disulfide bridges. It is also the place where nascent polypeptides are glycosylated – the most common post-traductional modification which is a crucial event during protein folding and quality control processes (Bieberich, 2014). The LRR-RLKs are part of the large family of plant proteins which are N-glycosylated and many N-glycosylation acceptor sequences are present in all ECDs. In some pattern recognition receptors and receptors involved in developmental processes, proteins with mutations at residues which will create misfolded proteins have been shown to be part of endoplasmic reticulum protein complexes and directed to degradation (Hong et al., 2008, 2009, 2012; Li et al., 2009; Nekrasov et al., 2009; Lee et al., 2011; Su et al., 2011; Huttner and Strasser, 2012; Sun et al., 2012; Park et al., 2013; Robatzek and Wirthmueller, 2013; Huttner et al., 2014). The significance of all the structural feature modifications which have been mentioned above are still mostly unknown but classic biochemical and cell biological studies (e.g., domain swapping among orthologs and/or targeted point mutations using CRISPR/Cas9) should help to explore their functions in details and will provide many novel insights into the molecular characterization of LRR-RLKs.

## Author Contributions

NC, CP, EG, and AD designed the study; GD and AD performed the LRR-RLK extraction; J-FD and AD performed the phylogenetic clustering; NC and IF performed the selection footprint analysis; IF, NC, J-FD, MB, and AD analyzed the data; J-FD, AD, NC, and IF wrote the article.

## Conflict of Interest Statement

The authors declare that the research was conducted in the absence of any commercial or financial relationships that could be construed as a potential conflict of interest.

## Footnotes

1 https://github.com/SouthGreenPlatform/rap-green

## Abbreviations

Cys-pair: cysteine-pair
ECD: extracellular domain
ICD: intracellular domain
KD: kinase domain
LRR: Leucine-Rich Repeat
LRR-RLK: Leucine-Rich Repeats Receptor-Like Kinase
MD: monocots dicots
MLD: malectin-like domain
OG: orthologous group
RLK: Receptor-Like Kinase
SG: subgroup
SP: signal peptide
TM: transmembrane domain.
