# Corrigendum: New Insights on Leucine-Rich Repeats Receptor-Like Kinase Orthologous Relationships in Angiosperms

**DOI:** 10.3389/fpls.2017.00916

**Published:** 2017-05-24

**Authors:** Jean-François Dufayard, Mathilde Bettembourg, Iris Fischer, Gaetan Droc, Emmanuel Guiderdoni, Christophe Périn, Nathalie Chantret, Anne Diévart

**Affiliations:** ^1^CIRAD, UMR AGAPMontpellier, France; ^2^INRA, UMR AGAPMontpellier, France

**Keywords:** LRR, receptor, kinase, angiosperms, phylogeny, orthologs

In the original article, the reference for the *Prunus persica* (PRUPE, peach) sequence genome was incorrectly written as (Ahmad et al., 2011). It should be (Verde et al., 2013). The authors apologize for this error and state that this does not change the scientific conclusions of the article in any way.

## Conflict of interest statement

The authors declare that the research was conducted in the absence of any commercial or financial relationships that could be construed as a potential conflict of interest.
